# Frontal Alpha EEG Asymmetry Before and After Positive Psychological Interventions for Medical Students

**DOI:** 10.3389/fpsyt.2018.00432

**Published:** 2018-09-11

**Authors:** Yuan-Yuan Xu, Zheng-Quan Feng, Yuan-Jun Xie, Jin Zhang, Shu-Hao Peng, Yong-Ju Yu, Min Li

**Affiliations:** ^1^Department of Military Psychology, Army Medical University, Chongqing, China; ^2^The Fifth Department of Daping Hospital & Surgery Institute, Army Medical University, Chongqing, China; ^3^Department of Biomedical Engineering, Army Medical University, Chongqing, China

**Keywords:** subjective well-being, positive psychological interventions, frontal alpha EEG asymmetry, emotion regulation, mental health

## Abstract

Subjective well-being (SWB) refers to traits concerned with happiness, fulfillment and enrichment and is a substantial predictor of a flourishing life. Interest in the promotion of well-being has blossomed in recent years, and it is widely reported that positive psychological interventions (PPIs) can effectively improve SWB. However, to date, the neural correlates of PPIs remain elusive. Since previous research has suggested that emotion regulation might be the theoretical foundation for potential working mechanisms, here we used electroencephalography (EEG) techniques to identify whether the intentional increase of subjective well-being through PPIs was associated with greater tonic left frontal activation. Fifty-five students met the inclusion criteria and were allocated to a randomized controlled trial that was single blinded. The intervention group received PPIs once a week for 10 weeks (*n* = 28). Meanwhile, students in a placebo control group (CG, *n* = 27) were asked to write down early memories every day for 10 weeks and were invited to share voluntarily at the weekly meeting. Measures of subjective well-being, depression and anxiety were assessed at pre-test and post-test. Forty-eight students completed the post-test, and the collected data were analyzed across time (PPIs, *n* = 25; CG, *n* = 23). It was found that students undergoing the 10-week PPIs reported larger improvement in SWB, and greater relief in self-rated depression and anxiety from pre-intervention to post-intervention than did those in the control group. As expected, in conjunction with the promotion of subjective well-being and the amelioration of emotional distress from pre- to post-treatment in the intervention group, a significantly increased coefficient of frontal alpha EEG asymmetry was found. In summary, these findings suggest that adaptive emotion regulation, which is characteristic of greater tonic left frontal activation, reflects the efficiency of PPIs and highlights the frontal alpha EEG asymmetry as a neural substrate linking PPIs and mental health.

Clinical Trial Registration Number: ChiCTR-ROC-17012636

## Introduction

Recent years have seen a blossoming interest in the field of human subjective well-being (SWB). SWB refers to a broad category of phenomena that include people's evaluations of their lives (e.g., “life is satisfactory”) and emotional responses (e.g., “I am happy”) (1). As a predominant positive psychological component, subjective well-being is considered to play a critical role in fostering the quality of people's social lives (1). According to Shin et al. (2), positive affect contributed to the promotion of peer acceptance, teacher-assessed adaptation, and initiating positive relationships with peers among children. As is revealed by Boehm and Lyubomirsky (3), the most popular and likable people are usually those more happier ones. In addition, there are a number of studies on the predictive power of SWB on health and longevity (4). For example, Xu and Roberts studied 6,856 participants from Alameda Countyin California over a period of 28 years (1966 to 1993). The results indicated that positive affects, satisfaction with life, and domain satisfactions are correlated with lower risk of all-cause and natural cause mortality (risk ratios from 0.90 to 0.99). Unnatural-cause mortality can also be predicted by positive affects and satisfaction with life (risk ratios from 0.86 to 0.96) (5). Just as Diener proposed, “subjective well-being is not only a desirable outcome but can also be an important predictor of future life outcomes” (1).

Considering the beneficial effects of SWB, intentionally cultivating SWB through well-being interventions can therefore serve as an antidote to negative emotions and motivates people to seek for rewards in their lives (6). In addition, the frequency of negative affect, behaviors, and thoughts, which are typical features of various risk factors leading to psychopathology, will thus be reduced (7). An ideal way to increase well-being is by means of positive psychological interventions (PPIs) (8, 9). According to Layous et al. “PPIs are treatment methods or intentional activities that aim to cultivate positive feelings, behaviors, or cognitions” (7). They are simple and easy to use, and most are self-administered, with participants accepting unified and normalized training instructions and performing the activities themselves (7). These intentional activities include: *writing about three good things that happened each day, making a gratitude visit* or *writing letters of gratitude, identifying signature strengths and using them in a new way* (10), *expressing gratefulness for life's blessings* that people experience each day (11), and *imagining the best possible self* (12), etc. All of these exercises underline the cultivation of personal character strengths shared by individuals with a high level of well-being (such as three *good things* [the strength of hope], *gratitude visit* [the strength of gratitude] and *counting kindness* [the strength of kindness]) (13). These diverse PPIs strategies have shown beneficial effects on subjective well-being in general (14, 15) and on symptoms of various mental disorders (16, 17).

The beneficial effects of PPIs are well recognized and certified, while more work needs to be done on the underlying mechanisms of PPIs, particularly the supporting empirical evidence. Considering the improvement in emotional status is one of the most noteworthy benefits of PPIs. It is argued by some researchers (18) that the cultivation of adaptive emotional regulation plays the key role in health promotion; and in their reports, emotion regulation strategies are proposed as the theoretical foundation of possible working mechanisms, which contain “situation selection, situation modification, attentional deployment, cognitive change, and response modulation” (19). A recent study conducted by Wellenzohn et al. (20) tested the proposed mechanisms. In this study, a random division was made among 695 adults, and all the participants were randomly assigned to one of “three funny things” intervention group, which needed they write down three funny things every day in a way to center on the present, the past, or the future, respectively, or a placebo control group (i.e., early memories exercises). The results indicated that the intervention could increase participants' well-being and decrease depression significantly. Moreover, compared with the placebo control group, the intervention groups were more capable in boosting savoring and turning the attentional deployment to the positive (20). This initial study provides the first support for the proposed working mechanisms of effective PPIs. However, studies of the neural correlates of PPIs are remain elusive.

Extensive electroencephalography (EEG) research has shown that emotion-related traits and states are associated with frontal EEG asymmetry and the manipulation of an individual's anterior alpha EEG asymmetry is associated with later emotion regulation and emotion perception (21). For example, Jackson and his colleagues based on 47 adults suggested that greater left superior frontal activation at pre-test was correlated with a lower startle magnitude after the offset of negative stimulus (22). In another EEG study (23), biofeedback training was randomly given to 18 right-handed female participants, which were designed to increase right frontal alpha relative to left frontal alpha (Group A) or in the opposite direction (Group B); before and after the 5-week intervention, three short emotionally evocative silent films (happy, neutral, and sad) were presented. The results demonstrated that participants in group B reported more positive emotions when they watched the happy films and expressed less corrugator activation when they watched the sad films (23).

Although a recent EEG study had found that there was a positive correlation between left-sided frontal activation and the levels of SWB (24), it is not clear whether the intentionally increase of subjective well-being through PPIs was associated with greater tonic left frontal activation. To address this gap in the literature, here we examined the frontal alpha activities before and after PPIs, and we hypothesized that the PPIs might boost an individual's mental well-being to the extent of the improvement of ability to properly regulate emotions, which manifested as an increased coefficient of frontal alpha EEG asymmetry.

## Materials and methods

### Participants

The study was advertised via a recruitment notice advertisement posted around a medical university. Medical students were chosen because they were believed a more “at risk population” for mental disorders (25). After applying for the training, participants were asked to fill out the screening questionnaires (*n* = 116). We applied the following inclusion criteria: (a) not having participated in any psychological intervention before the training, (b) not participating in any other psychological intervention during the study, (c) being right-handed individuals, (d) having no history of neurological or psychiatric problems, and (e) having normal visual acuity or corrected visual acuity. Students who met all the inclusion criteria were then offered detailed information about the research target and process. Written informed consent was obtained from all subjects before the intervention. The Satisfaction with Life Scale (SWLS), the Positive Affect, and Negative Affect Schedule (PANAS), the Center for Epidemiological Studies Depression Scale (CES-D), and the State Anxiety Inventory (SAI) were adopted to assess their levels of subjective well-being, depression and anxiety.

Following the provision of signed consent and the completion of the screening questionnaires and pre-assessment, participants (*n* = 56) who met all the inclusion criteria were randomly assigned to the intervention condition (*n* = 28) or to the placebo control condition (*n* = 28) by an online true random-number service independent of the investigators. The regional ethics committee of Army Military Medical University approved this consent procedure and the study protocol.

### Procedure

Participants in the intervention group and the control group received strength-based PPIs or the placebo interventions, respectively. To certify the beneficial effects of PPIs on subjective well-being, self-report measures were conducted on the internet before and immediately after the interventions. The programme was set as an optional course named “Psychology and Life” so that the delivery of the programme could fit within the school term and study objectives. Both ten-session interventions were administered in group format by three school psychologists. Although the procedure requires skilful manipulation and was not blinded to the practitioners, students were blinded to the group allocation; thus, the study was a randomized single-blinded control trial.

**Figure 1:**
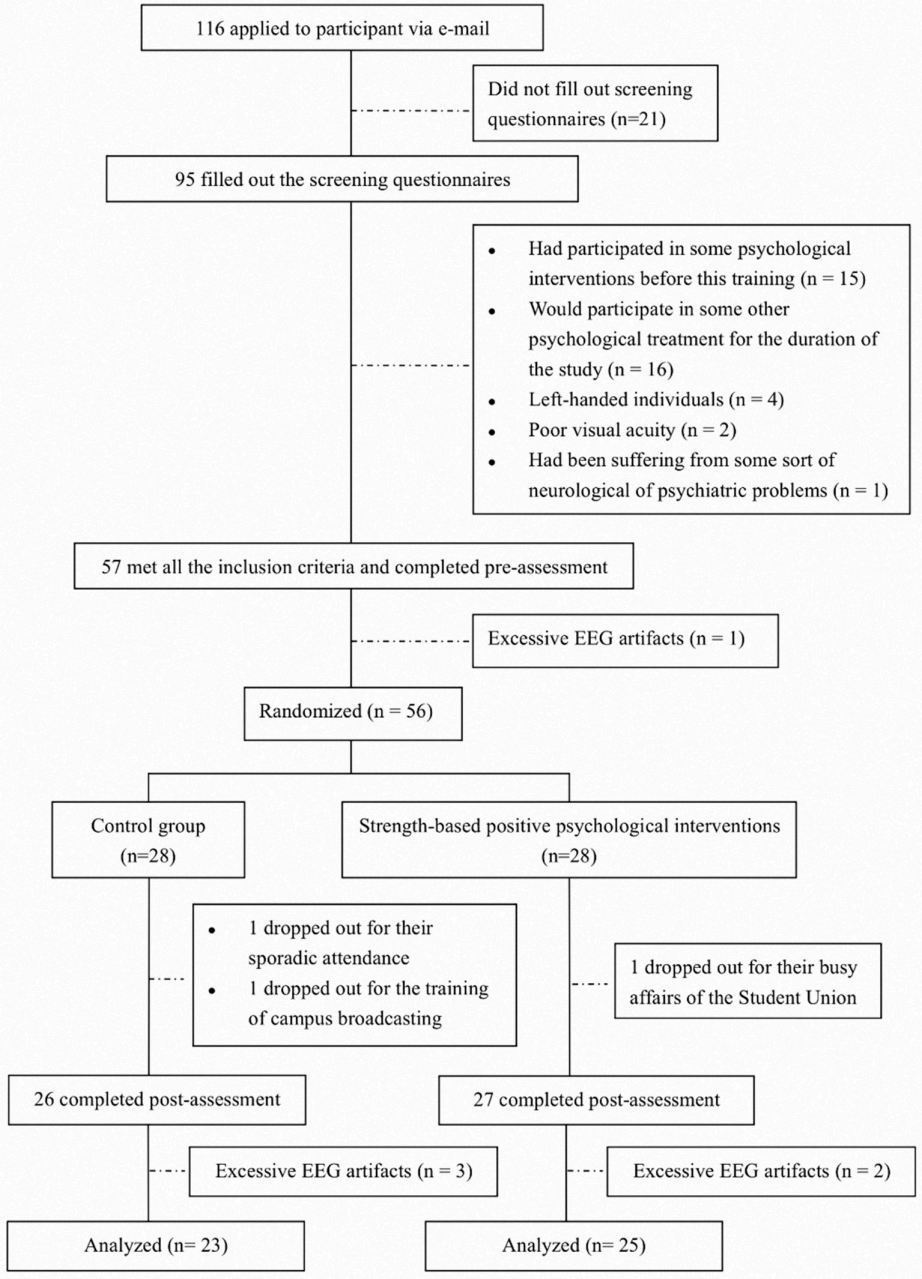
Flow of participants.

In addition, students were asked to undergo EEG recordings before and after training (see flow chart in Figure 1). For the EEG assessment, students were instructed to sit in a sound-attenuated room and to keep two 2-min intervals for eyes open and for eyes closed respectively in a counterbalanced order across participants. In the eyes open intervals, participants need to have their eyes fixed on a white cross, which was displayed in the center of a computer monitor.

### The well-being intervention

Weekly sessions for 10 consecutive weeks were offered. The first session consisted of an introduction to the intervention purpose, in addition to some activities designed to help to enhance participants' interactions and establish a friendly and supportive group environment. Sessions two through nine were structured as 3 parts in line with Seligman's framework (10) and with Suldo's (26) intervention procedures for boosting well-being through intentional activities, consisting of increasing 1. grateful expressions for past events (sessions 2 and 3), 2. satisfaction by means of creative use of significant character strengths (sessions 4, 5, and 6), and 3. positive future-oriented emotions by cultivating a hopeful, optimistic and goal-directed thinking habit (sessions 7, 8, and 9) (26). The last session was centered on reflecting on the practice of learned skills in daily life, including a review of activities and the theoretical framework for increasing well-being. In brief, this 10-week, 2-h-per-week intervention consisted of the following exercises: introduction to gratitude and gratitude letter-writing, gratitude visit, acts of kindness (27), identifying signature strengths (10), use of signature strengths in new ways and savoring (10), stopping worrying (28), three good things, and the best possible self (12), in addition to an introductory (the first meeting) and concluding session (the last meeting) (see Supplementary Table 1).

#### Positive interpretations of past events

The crucial purpose of sessions 2 and 3 was to cultivate a gratitude framework for the explanation of past events. A gratitude-related video was shown as a method of revealing the importance of expressing gratitude and guiding students to understand the correlation between gratitude and well-being. The exercises of the gratitude letter and gratitude visit were administered to focus participants' thoughts on grateful people and events, and to increase the experience of gratitude and subjective well-being by strengthening the association between thoughts, feelings, and actions (26). Students were asked to compose a gratitude letter and send the letter to someone (far away from them) whom are extremely grateful, but whom they have never properly thanked; visit someone near them who is extremely compassionate.The students were encouraged to share the experience of their gratitude practice at the next session.

#### Positive experience of present events

“Act of kindness,” “identify signature character strengths,” and “use signature character strengths creatively” were collected into this topic to promote engagement and satisfaction in daily life. The acts of kindness exercise required that participants do something good (that is, to practice beneficence) and to record the experience (27). From the perspective of emotion regulation, this could influence the students' choices of what situations they might enter and further affect the resulting emotional experience. In addition, participants were invited to identify their signature character strengths through the online Values in Action Inventory of Strengths for Youth (VIA-Youth) (29), and to try to use their top 5 signature strengths in new and different ways. To intensify and savor the experience of flow and satisfaction derived from this activity, the students were required to record the feeling of creatively using these top strengths every day for 1 week.

#### Positive framework of future events

The main aim of sessions 7–9 was to cultivate a positive framework for consideration of future events. The worry journals were used to change students' existing irrational or negative beliefs and to build reasonable cognition. Students were asked to report the records of “the things you worry about” that they had written down previously and to count how many of these concerns had truly happened (28). The exercise of three good things was intended to develop learned optimism. According to Seligman (10), the act of recording 3 pleasant things was capable of helping participants develop positive attitudes and get into the habit of being aware of the bright aspects of surrounding events, which enabled individuals to stay open-minded in a dilemma and effectively address the predicament. The “best possible self” exercise (12) required students to set a short-term goal and make a practical plan to achieve the goal; according to Snyder's hope theory, the detailed plan must contain ➀possible obstructions; ➁paths to overcome difficulties and ➂motivations for success. In addition to promoting the students' feeling of hope and their resulting sense of well-being, detailed goal mapping could reduce the uncertainty of the future, which was the main risk factor leading to anxiety disorders (30).

### The placebo control condition

Participants in the control group received no instruction for the intervention programme. Instead, they were asked to write down something from their early memories every day for 10 weeks and were invited to share voluntarily at the weekly meeting.

### Self-report measures and analyses

The intervention was assessed in terms of related changes with the participants' subjective well-being, as well as in terms of traditional negative indicators of mental health (i.e., depression and anxiety), so that the secondary effects of the intervention on psychopathology could be explored.

#### Subjective well-being

Subjective well-being (SWB) is abroad concept that includes experiencing more positive emotions, a high degree of satisfaction with life and low levels of negative moods (31). A measure of SWB was then constructed with the Positive Affect and Negative Affect Schedule (PANAS) (32) and with the SWLS (33) (i.e., PA+SWLS–NA). The PANAS has 20 adjectives that indicate positive and negative affect. A five-point Likert scale ranging from 1(very slightly or not at all) to 5 (extremely) was used to evaluate participants′ mood state during a certain time frame. The internal consistency coefficient of the positive affect subscale and the negative affect subscale for our sample were 0.86 and 0.83, respectively. The SWLS is a 5-item self-report questionnaire. Participants need to evaluate the degree to which they agree or disagree with items such as “In most ways my life is close to my ideal” on a seven-point Likert scale range from 1 (strongly disagree) to 7 (strongly agree). The internal consistency coefficient of SWLS for our sample was 0.96.

#### Mental health

Depression was assessed using the Center for Epidemiological Studies Depression Scale (CES-D) (34). Subjects are invited to rate the frequency of occurrence of the symptoms from 0 to 3 (0–occasionally or never, 1—sometimes, 2—often or half of the time, and 3—mostly or continuously). Total scores ranged from 0 to 60, with lower scores indicating less depression. The internal consistency coefficient for our sample was 0.91.

The SAI (35) was administered to assess anxiety. SAI is a 20-item self-report questionnaire designed to measure certain subjective feelings such as nervousness, worries, and fear at present or during a specific period of time. Each question is rated on a four-point Likert scale (1 = not at all, 4 = very much so), with total scores ranging from 20 to 80. The Cronbach alpha estimate for our sample was 0.85.

#### Statistical analyses

All analyses were conducted using SPSS19.0. Independent samples *t*-tests and Chi-square tests were conducted to explore the homogeneity of the intervention group and the control group. We conducted repeated measures ANOVA on outcome measures with test time as a within-group factor and condition as a between-group factor. A *p*-value of < 0.05 with an α-level of 5% was set for statistical significance. Partial η-square values (η_*p*_^2^)according to Cohen (36) were calculated for estimating effect sizes.

The purpose of these analyses was to determine the benefits of the intervention on students' well-being, depression and anxiety in the two conditions.

### Eeg recording, reduction, and analysis

Tin electrodes attached to a stretch-lycra electrode cap were used to record EEG for an 8-min resting period consisting of two eyes-open parts and two eyes-closed parts with a counterbalanced sequence, each 2 min in length. Horizontal electro-oculographic activity was recorded from the epicanthus of each eye and vertical from the supra-and infra-orbital positions of the right eye. Continuous EEG data were collected through a 64-channel NuAmp acquisition system (Neuroscan Inc.) using the International 10–20 system, with a reference at Cz and the area between Fz and Fpz as the ground. All electrode impedances were below 5 kΩ. Offline, a digital average mastoid reference, (M1+M2)/2, was performed.

After the raw EEG data were manually scored for eye movement and muscle artifact, the EEG was reconstructed into non-overlapping epochs of 2 s. After artifact detection, for each channel of each participant, the power spectrum was derived from the Fast Fourier Transform and Hamming window every 2 s in each baseline type (eyes open, eyes closed); thus, each 2 min baseline type contained 60 non-overlapping 2-s epoch, and EEG findings were specific to both open and closed eyes. The average power spectrum for each 2-min period was obtained. The total power in the alpha band (8–13 Hz) was extracted and were then averaged across minutes. In order to obtain normalized data, the power values were natural logarithm (ln) transformed. A measure of frontal EEG asymmetry (i.e., ln [right]–ln [left]) was then derived for the homologous scalp site of interest. Since alpha power is usually considered as inversely proportional to cortical activity, lower value of the index reflects the less left side activity (37).

## Results

### Descriptive statistics

Figure 1 presents the subjects' flow chart. Among all the participants, 116 (47 female, mean age = 21.63 ± 3.14) applied for the training. Of the students, 55 were eligible to join in the research and were randomly allocated into the treatment and placebo control groups. Post-test follow-up failed to be made in 7 students causing a 12.73% drop-out rate. Table 1 shows students' demographic data. According to the results of independent samples *t-*tests and chi-square tests, no significant difference in the demographic variables was found.

**Table 1:** Comparisons between the groups regarding demographic data.

	**Intervention group** ***n*** = **25**		**Control group** ***n*** = **23**		**Total** ***n*** = **48**	
	**Mean**	**SD**	**Mean**	**SD**	**Mean**	**SD**
**Age**	22.14	1.55	21.92	1.98	22.03	1.75
	***n***	**%**	***n***	**%**	***n***	**%**
**GENDER**						
Male	10	40.00	11	47.83	21	43.75
Female	15	60.00	12	52.17	27	56.25
**FAMILY STRUCTURE**						
Single parent	7	28.00	3	13.04	10	20.83
Two parent	15	60.00	14	60.87	29	60.42
Other conditions	3	12.00	6	26.09	9	18.75
**ORIGIN FROM CITY OR COUNTRY**						
City	9	36.00	13	56.52	22	45.83
Country	16	64.00	10	43.48	26	54.17

*Using Independent samples t-tests and Chi-square tests, there was no significant difference in the demographic variables between the two groups*.

### Changes in self-report measures

In Table 2, the baseline value of the self-report measures for the intervention group and placebo control group are depicted. No significant group differences at pretest were found on any measure.

**Table 2:** Means (*M*), Standard Deviations (*SD*) for subjective well-being, depression and anxiety at T1, T2 for the intervention group (*n* = 25) and control group (*n* = 23), and the results of the repeated measures ANOVA after data were split by group.

		**T1 *M (SD)***	**T2 *M (SD)***
SWB	PPIs	35.84 (15.94)^a^	43.48 (9.23)
	CG	38.00 (12.63)	36.09 (12.18)
CES-D	PPIs	17.72 (8.56)^a^	11.84 (8.79)
	CG	17.74 (8.99)	18.27 (6.43)
SAI	PPIs	36.56 (13.14)^a^	31.72 (10.33)
	CG	36.04 (9.97)	38.43 (8.64)
F8-F7	PPIs	−0.06 (0.18)^a^	0.20 (0.12)
	CG	−0.01 (0.50)	−0.09 (0.78)
F4-F3	PPIs	0.07 (0.13)^a^	0.34 (0.72)
	CG	0.16 (0.48)	0.07 (0.13)
FC6-FC5	PPIs	−0.05 (0.23)	0.07 (0.21)
	CG	0.01 (0.44)	−0.18 (0.66)
FC4-FC3	PPIs	−0.05 (0.17)^a^	0.20 (0.33)
	CG	−0.06 (0.49)	−0.10 (0.42)

*PPIs, positive psychological interventions; CG, control group; T1, pre-intervention; T2, post-intervention; SWB, Subjective Well-being; CES-D, Center for Epidemiological Studies Depression Scale; SAI, State Anxiety Inventory*.

a *p < 0.05 for repeated measures ANOVA post hoc test for T1-T2*.

The results of the repeated measurement ANOVA after splitting the data to analyse PPIs and control group across time (T1–T2) are included in Table 2. The table shows that mean levels of subjective well-being increased numerically over the course of time in the intervention group, whereas only subtle changes were observed in the control group. In respect to emotional distress, the intervention participants reported statistically significant decreases in depression and anxiety from T1 to T2, while no significant change was observed in the control group.

Repeated measurement ANOVA results are detailed in Table 3. Results showed that within-subject effect of time was significant for SWB but not for anxiety or depression. In addition, there were significant interactions between time and group in terms of SWB, SAI, and CES-D. Further simple effect analysis indicated significant training effects at post test for subjective well-being [*F*_(1, 46)_ = 5.67, *p* = 0.02, η_*p*_^2^ = 0.11], anxiety [*F*_(1, 46)_ = 5.91, *p* = 0.02, η_*p*_^2^ = 0.11], and depression [*F*_(1, 46)_ = 8.22, *p* = 0.01, η_*p*_^2^ = 0.15], with a higher level of well-being and a lower level of depression and anxiety in the intervention group. The results indicated that the strength-based PPIs could contribute to promoting an increase in the individuals' well-being and mitigating mental distress.

**Table 3:** Repeated measurement analysis of variance on groups (the training group and control group), and time periods (pre-test, post-test) for subjective well-being, depression and anxiety.

	***N***	**Within subject effect**			**Time** × **Group**			**Between subject effect**		
		***F***	***p***	***η_*p*_*^2^**	***F***	***p***	***η_*p*_*^2^**	***F***	***p***	***η_*p*_*^2^**
SWB	48	5.32	0.03	0.10	14.83	<0.01	0.24	0.57	0.45	0.01
CES-D	48	3.53	0.07	0.06	5.04	0.03	0.10	2.82	0.10	0.06
SAI	48	1.21	0.28	0.03	10.57	<0.01	0.19	1.15	0.29	0.02
F8-F7	48	0.99	0.33	0.02	3.54	0.07	0.07	1.36	0.25	0.03
F4-F3	48	0.41	0.53	0.01	5.09	0.03	0.10	1.97	0.17	0.04
FC6-FC5	48	0.16	0.69	<0.01	2.74	0.11	0.06	1.47	0.23	0.03
FC4-FC3	48	2.22	0.14	0.05	4.12	0.04	0.08	3.93	0.06	0.07

*SWB, Subjective Well-being; CES-D, Center for Epidemiological Studies Depression Scale; SAI, State Anxiety Inventory*.

### Change of frontal alpha EEG asymmetry

The means and standard deviations of the following four alpha asymmetry scores in the PPIs and CG before and after the intervention are presented in Table 2: lateral frontal (F8-F7), frontal (F4-F3), fronto-central (FC4-FC3, FC6-FC5) (38). Using the *t*-test, alpha EEG asymmetry baseline measures were not significantly different between the intervention group and the control group. Repeated measurement ANOVA results were detailed in Table 3. Results indicated that there was no main effect of time and no main group effect. Significant time (T1 and T2) by group (PPIs and CG) interactions were found for F4-F3 alpha asymmetry and FC4-FC3 alpha asymmetry. Further simple effect analysis indicated significant training effects at post test for F4-F3 alpha asymmetry [*F*_(1, 46)_ = 4.51, *p* = 0.04, η_*p*_^2^ = 0.09] and FC4-FC3 alpha asymmetry [*F*_(1, 46)_ = 8.49, *p* = 0.01, η_*p*_^2^ = 0.16], with a more positive asymmetry score in the intervention group.

### Correlations between frontal EEG asymmetry change and behavioral change

According to Table 4, the control group exhibited no significant correlations between behavioral changes and EEG changes. However, significant correlations were found between changes of self-reported outcome measures (i.e., subjective well-being and anxiety) and changes of frontal alpha asymmetry (i.e., F4-F3 and FC6-FC5) in the intervention group. In addition, the PPIs sample also exhibited significant negative correlation between change of depression and change of F4–F3 alpha EEG asymmetry. For all students, changes of F4-F3, F8-F7, FC4-FC3, and FC6-FC5 frontal EEG asymmetry were negatively related to changes of CES-D and SAI scores (only the correlation with change of F4–F3 alpha EEG asymmetry was significant) and were positively related to the change of SWB scores.

**Table 4:** Bivariate correlations between changes of self-reported outcome measures and changes of frontal EEG asymmetry for each group and across all participants.

**Group**	**Measures**	**Δ(F8-F7)**	**Δ(F4-F3)**	**Δ(FC4-FC3)**	**Δ(FC6-FC5)**
PPIs (*n* = 25)	ΔSWB	0.26	0.77^**^	0.30	0.53^**^
	ΔCES-D	−0.15	−0.81^**^	−0.28	−0.34
	ΔSAI	−0.36	−0.54^**^	−0.12	−0.53^**^
CG (*n* = 23)	ΔSWB	0.30	0.05	0.26	0.22
	ΔCES-D	−0.33	−0.26	−0.22	−0.34
	ΔSAI	−0.15	−0.11	−0.14	−0.20
All (*n* = 48)	ΔSWB	0.33^*^	0.57^**^	0.35^*^	0.29^*^
	ΔCES-D	−0.32^*^	−0.61^**^	−0.29^*^	−0.34^*^
	ΔSAI	−0.26	−0.42^**^	−0.23	−0.27

*ΔSWB, ΔCES-D, ΔSAI, Δ(F8-F7), Δ(F4-F3), Δ(FC4-FC3), Δ(FC6-FC5) represent the discrepancy of subjective well-being, depression, anxiety, and frontal alpha EEG asymmetry between the baselines and post-test (T2-T1), respectively*.

* p < 0.05,

** *p < 0.01*.

## Discussion

This study aimed to explore the impact of the PPIs, compare with a placebo control, upon subjective well-being and emotional distress, as well as on the corresponding neural electrical changes. Self-reported results showed that the intervention had significant effects on subjective well-being, depression, and anxiety. First, the data indicated that students participating in the10-week PPIs reported a greater increase in subjective well-being compared to the control participants. Over the 3 months, subjective well-being increased immediately at post-test in the intervention condition, while no significant change was found in the placebo control group. The results parallel the positive correlation documented between well-being and coping capacity and resilience (39) and suggest us that through school interventions, the promoting of well-being could thus improve students' mental toughness, coping styles, and developmental processes (40). Moreover, Ryff et al. proposed that well-being has a meaningful defensive function in aspect of sensitivity to chronic and acute life stresses (41). Consistent with this, participants in the intervention group showed greater relief in depression and anxiety relative to students in the placebo control group from pre- to post-test. The observed effects are worth noting as medical students tend to experience more distress in the middle of the semester, during which exams and assignments come in great number (42). The results thus certify the viewpoint that the PPIs can play an important role if conducted in the middle or the end of the semester, when students are in a more vulnerable psychological state (43).

Although much attention has been directed toward the improvement of human subjective well-being, the neurobiological mechanisms underlying these well-being interventions remain elusive. On the base of Gross's emotion regulation-model, Quoidbach et al. (18) frame different kinds of PPIs and suggested emotion regulation strategies to be the theoretical foundation for potential working mechanisms (20). Based on this, the current study proposed that PPIs might promote a participant's mental well-being to the extent that the ability to properly regulate emotions improved, which was manifested as an increased coefficient of frontal alpha EEG asymmetry. The results align with the initial hypothesis: In conjunction with the promotion of subjective well-being and the amelioration of emotional distress from pre- to post-treatment in the intervention group, obvious electrocortical changes occurred typically with a shift to the greater rest left frontal alpha EEG asymmetry.

According to Beck's generic cognitive model (GCM), individuals with emotional distress are tend to pay more attention to mood—congruent stimuli (44), which facilitates biased interpretation and biased memory, this, in turn, leads to the formation of biased cognitive processing schemas or beliefs (45). In addition, the activated schema could initiate further affective, motivational, and behavioral responses and trigger the downward spirals to mental disorders (39). Previous research has proposed that momentary thought–action repertoires will be broaden by positive emotions, which could widen the thoughts and actions that pass through the mind (6), and thereby promoting more creative and flexible cognitive associations and appraisals to face emotional situations (46), and create the urge to sit back and enjoy current life circumstances or stimulate people to act and approach rewards in their lives (24). Since the way in which we consider and reflect to the world shapes what we experience, the broadened attention to positive information, a more flexible cognitive association and active coping strategies toward improved well-being (6) could have an adaptive emotion-regulating effect in everyday emotional situations; this would enable people to place the life events in a broader context, reducing the adverse effects of any typical negative event, and thus, countering the downward spirals of negativity (6, 39).

Moreover, benefiting from the scope of attention and cognition broadened by positive emotions, peoples' lasting coping resources would also be augmented through enhancing flexible and creative thinking ability (6); such resources would include lasting social bonds and attachments prompted by play, enduring physical resources promoted by exploring, and improved self-insight and advanced world view prompted by savoring (6). With regards to our intervention, the positive activities such as the gratitude visit, which asked participants to express their appreciation to someone who had done something good for them, or the act of kindness, which required the students to perform a good work and record their feelings, could strengthen interpersonal relationships and optimize their social support networks. When people later faced threatening situations, these greater personal resources would have translated into more adaptive strategies, and in turn, more rational emotional and behavioral responses (6, 39, 45). This virtuous circle could contribute to triggering upward spirals of well-being toward greater adaptation and mental health (39, 47). Taken together, the prefrontal activation in the intervention group is identified to activate the regulatory structures ascribing to the broader, flexible and positive attentional, cognitive, and behavioral repertories.

Some limitations of the current study should be mentioned. One point of concern is the small sample size. The findings were based on 48 medical students, which possibly undermined the significance of several interactive and simple effects, so a larger sampling is needed to replicate these results in future studies. Second, the dropout rate in this study was 12.73%; in order to increase the overall response rate and keep students motivated to remain in the study, upcoming studies might offer more ways to include more students, such as monetary incentives or credits reward. Another limitation of this study is its single-blind nature. A large population study that is blind to both participants and practitioners and long-term follow-up must be considered. Finally, in addition to recording the frontal alpha activity at rest before and after the well-being intervention, future studies should detect participants' continuous brain electrical activities as they process emotional stimuli; this could help uncover the possible effects of subjective well-being on different stages of emotion regulation, such as initial attentional bias, later cognitive process, and behavioral responses.

## Conclusion

Above all, the data from this study have shown that strength-based PPIs can effectively improve young adults' subjective well-being and mitigate the symptoms of depression and anxiety. Crucially, the process of adaptive emotion regulation might be the potential mechanisms by which PPIs brought benefits. Our findings may have clinical implications for providing potential biomarkers for the early prevention of mental disorders. Furthermore, beyond psychological interventions related to well-being, the neurofeedback training of subjective well-being can be developed to improve levels of health.

## Data availability statement

The datasets Analysis data for this study can be found in the Baidu Netdisk https://pan.baidu.com/s/12E7Bw0EBabFXhAPT3hrXdw.

## Ethics statement

This study was carried out in accordance with the Declaration of Helsinki. The protocol was approved by the regional ethics committee of Army Military Medical University. All subjects gave written informed consent in accordance with the Declaration of Helsinki.

## Author contributions

Y-YX and ML contributed to the design and conception of this study. Y-YX, Z-QF, and Y-JX elaborated the study protocol and gained ethical approval. JZ, Y-JY, and S-HP developed the intervention program. Y-JX wrote the manuscript, and Z-QF, Y-JX, JZ, and S-HP complemented the description of the design, randomization, and the statistical analysis. Z-QF, Y-JY, and ML participated in a critical review of the manuscript. All authors have contributed to the revision of the initial manuscript and have read and approved of the final version of the article submitted.

### Conflict of interest statement

The authors declare that the research was conducted in the absence of any commercial or financial relationships that could be construed as a potential conflict of interest.
