# Supplementary material for: Frontal Alpha EEG Asymmetry Before and After Positive Psychological Interventions for Medical Students
Source: Front Psychiatry. 2018 Sep 11;9:432. doi: 10.3389/fpsyt.2018.00432 (PMC6141827; doi:10.3389/fpsyt.2018.00432)
Supplement: Supplementary file 1 [file Table_1.DOCX]

Supplementary Material

Positive psychological intervention promotes subjective well-being and may be accompanied by changes in frontal alpha asymmetry

Yuan-Yuan Xu^1^, Zheng-Quan Feng^2,3^, Yuan-Jun Xie^1^, Jin Zhang^1^, Shu-Hao Peng ^3^, Yong-Ju Yu^1^, Min Li^1^*

*** Correspondence:** Dr. Min Li: limin52267@tmmu.edu.cn

# Supplementary Table

| **Supplementary Table 1** Idealized session by session description of strength-based positive psychological interventions | | |
| --- | --- | --- |
| Session and topic | Goal | Description |
| 1: Introduction | Introduce the strength-based positive psychological intervention format and purpose  Establish friendly and supportive group atmosphere | Get to know each other exercise: try to find congenial partner use non-verbal communication  Develop into a group and discuss the key group rules, as appropriate for the client group and context  Clarify the framework and purpose of intervention (i.e., increase well-being through intentional activities)  Homework: Each evening, write down ①the things you worry about, ②the kindness you express |
| ***Positive emotions about the past*** | | |
| 2: Gratitude introduction and gratitude letter | Define gratitude and discuss the benefits of feeling grateful and expressing gratitude  Rate students’ gratitude  Learn to express gratitude | Watch a gratitude-related video, and discuss: why may gratitude be important  Rate student’s current level of gratitude and well-being  Gratitude letter exercise: students are encouraged to think of someone (far away from them) to whom they are extremely grateful, but who they have never properly thanked, compose a gratitude letter to the person and send the letter to them |
| 3: Gratitude visit | Make connection between gratitude and well-being  Learn more directly method of expressing gratitude to boost well-being | Gratitude timeline exercise: think about people in their lives to whom they are grateful, and identify which of their actions in particular deserve recognition  Rate student’s current level of gratitude and well-being, and discuss the connection between grateful thoughts and positive feelings  Gratitude visit exercise: students are encouraged to visit someone around them who are extremely merciful |
| ***Positive emotions within the present*** | | |
| 4: Act of kindness | Define kindness and how it can impact well-being  Detect students’ current frequency of kind acts  Learn method of using kindness to create a focus on positive appraisement of present events | Watch a kindness-related video  Students are encouraged to review diaries to explore the frequency of their kind acts  The role of kindness in creating a focus on positive interpretation is discussed  Homework: performing acts of kindness |
| 5: Identify signature character strengths | Define character strengths and how it can impact well-being  Assess students’ signature character strengths | Review homework: performing acts of kindness  Assessment of signature strengths via online completion of VIA-youth  Students are asked to sit in the middle of the group one by one to be pointed out their signature strengths by other participants in detail (highlight the actual concrete evidence).  Homework: use those signature strengths more in daily life |
| 6: Use of signature character strengths | Learn creative method of using strengths in daily life  Plan uses of signature strengths in new ways across life domains (school, friends, family) | Review homework: use signature strengths more in daily life  Students are encouraged to think of creative ways to use their signature strengths and develop individualized plans for new uses of signature strengths  Homework: use signature strengths in new ways |
| ***Positive emotions about the future*** | | |
| 7: Optimism | Identify and change negative expectations of the unknown  Define optimistic thinking and how it can impact well-being  Cultivate optimism about future and self-efficacy | Review homework: use signature strengths in new ways  Students are encouraged to share the records of “the things you worry about” that they have written down previously, and count how many of these worrying things have really happened.  Learn methods for developing an optimistic cognitive style  Homework: list three good things experienced over the past day |
| 8: Three good things | Make connection between using optimistic thinking and well-being | Review homework: three good things experienced over the past day  The role of good and bad memories is discussed in terms of maintenance of well-being as related to the future.  Students are encouraged to share the records of “three good things” they have written down previously, and conclude why they happened and how to increase the occurrence of these good things in the future. |
| 9: The best possible self | Define hope and how it can impact well-being  Learn method for using hope by envisioning goals, paths to active goals, and motivation for success | Students are asked to define hope and how it can impact well-being, and then to write about the best possible self in the future, imagining any possible obstructions, paths to overcome difficulties and motivations for success.  Visualize the best possible self. |
| 10: Integration | Review theoretical framework of intervention and exercises learned in the group  Encourage students to continue practice the helpful exercises | The overall framework of the training is reviewed  Students are invited to share their impression of the interventions  Progress is reviewed, and gains and maintenance are discussed  Some advice from a therapist is given. |

**
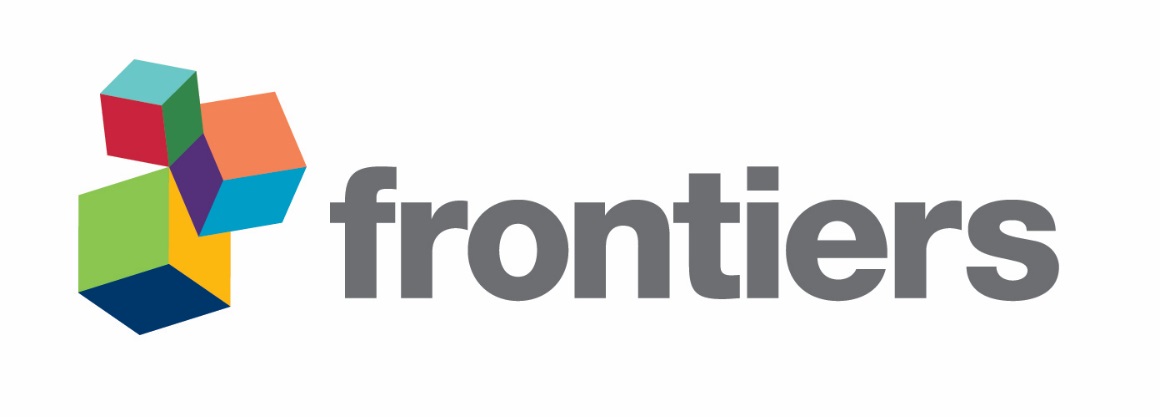
**
